# Palliative Stenting for Gastric Outlet Obstruction Secondary to Pancreatic Adenocarcinoma

**DOI:** 10.7759/cureus.53186

**Published:** 2024-01-29

**Authors:** Dhruv Patel, Furqan A Bhullar, Ariana R Tagliaferri, Gabriel Melki, Matthew A Grossman

**Affiliations:** 1 Internal Medicine, St. Joseph's Regional Medical Center, Paterson, USA; 2 Interventional Gastroenterology, St. Joseph's Regional Medical Center, Paterson, USA

**Keywords:** end of life and hospice care, interventional gastroenterology, palliative stenting, gastric outlet obstruction, pancreatic adenocarcinoma

## Abstract

Pancreatic cancer is one of the most fatal malignancies due to its advanced stages at the time of presentation. Often, it is only diagnosed when patients present with late-stage complications, such as gastric outlet obstruction (GOO). Many patients experience a poor quality of life due to the side effects of GOO, such as persistent nausea, vomiting, and an inability to tolerate an oral diet, and as such, patients deteriorate quickly after their diagnosis. Because pancreatic cancer is diagnosed at advanced stages, many patients are not surgical candidates, and thus treatment is tailored for palliative measures. With GOO specifically, gastrojejunostomy has been the mainstay of palliative management; however, endoscopic stent placement is a new, innovative, and minimally invasive alternative option. Herein, we present a case of GOO as a complication of pancreatic adenocarcinoma, treated with palliative endoscopic stent placement. Further research is warranted to identify patients who would most benefit from this modality of palliation in the treatment of advanced pancreatic cancer.

## Introduction

Gastric outlet obstruction (GOO), which inhibits gastric emptying, can be due to mechanical or motility etiologies [1]. Motility causes are most often due to gastroparesis, but gastric volvulus and gastroenteritis are also other etiologies [1-3]. Obstruction secondary to malignancy is the most common cause of the mechanical etiologies of GOO [1]. The most common malignancy causing mechanical GOO is gastric cancer, but pancreatic and duodenal cancers are also causative [2-3]. Formerly, GOO was more prevalent in patients with peptic ulcers in the setting of *Helicobacter pylori* infection. However, with the discovery of proton pump therapy, 50-80% of cases are associated with underlying malignancy [2-3].

Patients with GOO from any cause typically present with abdominal pain, weight loss, nausea, and/or vomiting, severely impacting the patient’s quality of life (QoL) [2]. These symptoms lead to further morbidities, including malnutrition, electrolyte abnormalities, and dehydration [1]. Extreme cases of GOO can also cause gastric perforation [4]. Historically, gastrojejunostomy, which is a surgical procedure to bypass gastric obstruction, was the standard of care to treat GOOs [5]. Although it carries a high success rate in relieving gastric obstruction, gastrojejunostomy is also associated with a morbidity of up to 40% due to complications such as perforation, bleeding, and secondary infections [6]. This procedure is also associated with higher mortality rates and prolonged hospital stays [5]. Moreover, up to 80% of patients are considered poor surgical candidates due to malnutrition, decreased chances of meaningful recoveries post-operatively, and a higher risk of intraoperative complications due to increased vasculature surrounding lesions and distant metastasis [7]. Thus, less invasive measures that offer rapid symptomatic relief in a palliative approach are pursued [7].

In the early 1990s, endoluminal stenting was introduced as an alternative approach to treating GOO [8]. Compared to the traditional palliative measure using surgical gastrojejunostomy, endoscopic stenting is associated with improved QoL, greater cost-effectiveness, shorter hospital stays, and decreased morbidity [5]. With favorable procedural outcomes, there are fewer adverse events, including renal failure and secondary infections such as sepsis and/or pneumonia [1-2]. Although published randomized trials have demonstrated a reduced risk of complications and a clear efficacy for patients with short life expectancies, there are no clear guidelines or indications for the use of endoscopic palliative stenting [1,3,9]. Moreover, there are no optimal criteria to select patients who would most benefit from this approach [10].

In this case report, we present a patient with end-stage pancreatic cancer who underwent palliative endoluminal stenting for the management of malignant GOO.

## Case presentation

**Figure 1:**
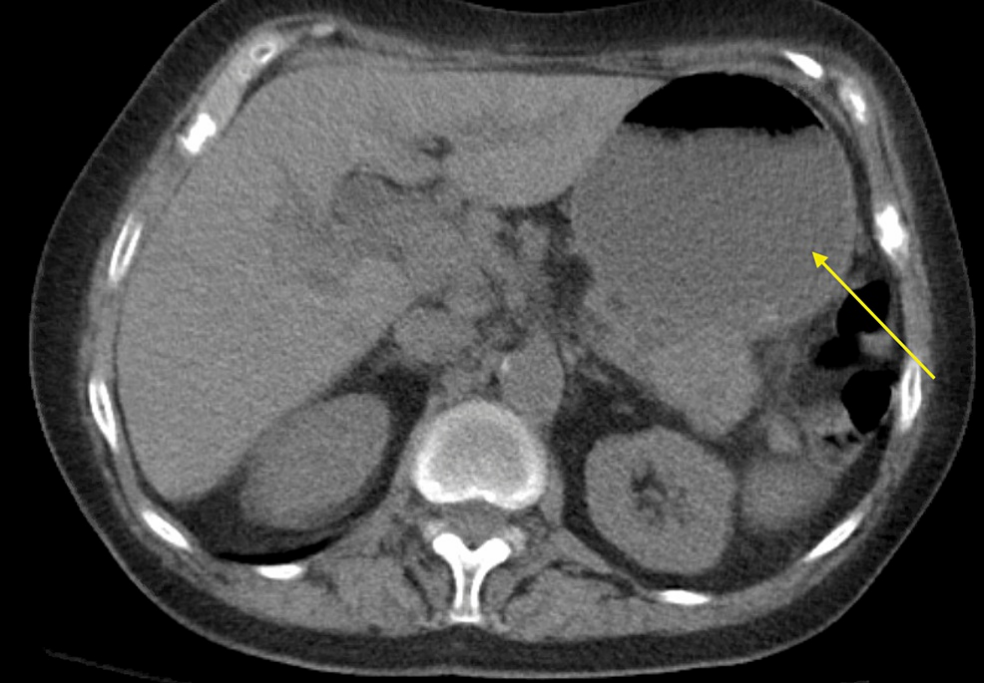
Computerized tomography (CT) of the abdomen and pelvis without contrast demonstrating gastric distension (yellow arrow) secondary to gastric outlet obstruction (GOO)

We present a 71-year-old cachectic Hispanic female with a past medical history of hypertension, asthma, anemia of chronic disease, and stage 4 pancreatic adenocarcinoma, which was diagnosed one year prior to presentation. She presented to the emergency department with severe epigastric abdominal pain, nausea, and projectile vomiting for one week prior to the presentation. She was unable to tolerate any oral intake but reported regular bowel movements. Her last chemotherapy session was one month prior to presentation, and at that time she had completed eight cycles of FOLFIRINOX (leucovorin calcium, fluorouracil, irinotecan hydrochloride, and oxaliplatin). On presentation, the patient was hypertensive with a blood pressure of 173/81 mmHg, a heart rate of 96 beats per minute, was afebrile, and was saturating 97% of oxygen on room air. On physical examination, she had dry mucous membranes with poor skin turgor. Her abdominal exam was significant for epigastric tenderness without rebound or guarding. She otherwise had normoactive bowel sounds in all quadrants without organomegaly or palpable masses. Laboratory results were significant for hypokalemic hypochloremic metabolic alkalosis and chronic normocytic anemia, consistent with the patient’s baseline (Table 1). Troponin was within normal limits. Twenty-eight days prior to this admission, she underwent a routine CT of the abdomen and pelvis, which demonstrated an interval increase in the pancreatic head mass by about 1.5 x 0.5 centimeters and evidence of new metastatic lesions in the liver. At this time, she was scheduled to continue the FOLFIRINOX chemotherapy. During this admission, a repeat CT of the abdomen and pelvis without contrast was performed, showing the stable appearance of the pancreatic tumor. The stomach appeared to be overly distended, suggesting GOO (Figure 1). A nasogastric tube was inserted for gastric decompression, and crystalloid fluids were administered.

**Table 1:** Complete blood cell count and comprehensive metabolic panel on admission

Laboratory parameters	Result	Reference range
White blood cell count	9.1	4.5-11.0x10^3^/mm^3^
Red blood cell count	3.59	4.0-5.33x10^6^/mm^3^
Hemoglobin	9.3	12.0-16.0 g/dL
Hematocrit	29.5	36.0-46.0%
Mean corpuscular volume	82.2	80.0-100.0 fL
Mean corpuscular hemoglobin	25.9	26.0-32.0 pg
Mean corpuscular hemoglobin concentration	31.5	31.0-37.0 g/dL
Red cell distribution width	15.9	0.5-16.5%
Platelets	271	140-440 K/mm3
Blood urea nitrogen	13	7-23 mg/dL
Creatinine	0.65	0.60-1.3 mg/dL
Estimated glomerular filtration rate	>60	>60 mL/min/1.73m^2^
Total bilirubin	0.6	0.3-1.1 mg/dL
Total protein	7.3	6.4-8.4 g/dL
Albumin	4.1	3.5-5.7 g/dL
Alkaline phosphatase	434	34-104 unit/L
Aspartate aminotransferase	19	13-39 unit/L
Alanine transaminase	22	7-52 unit/L
Lipase	4	11-82 unit/L

**Figure 2:**
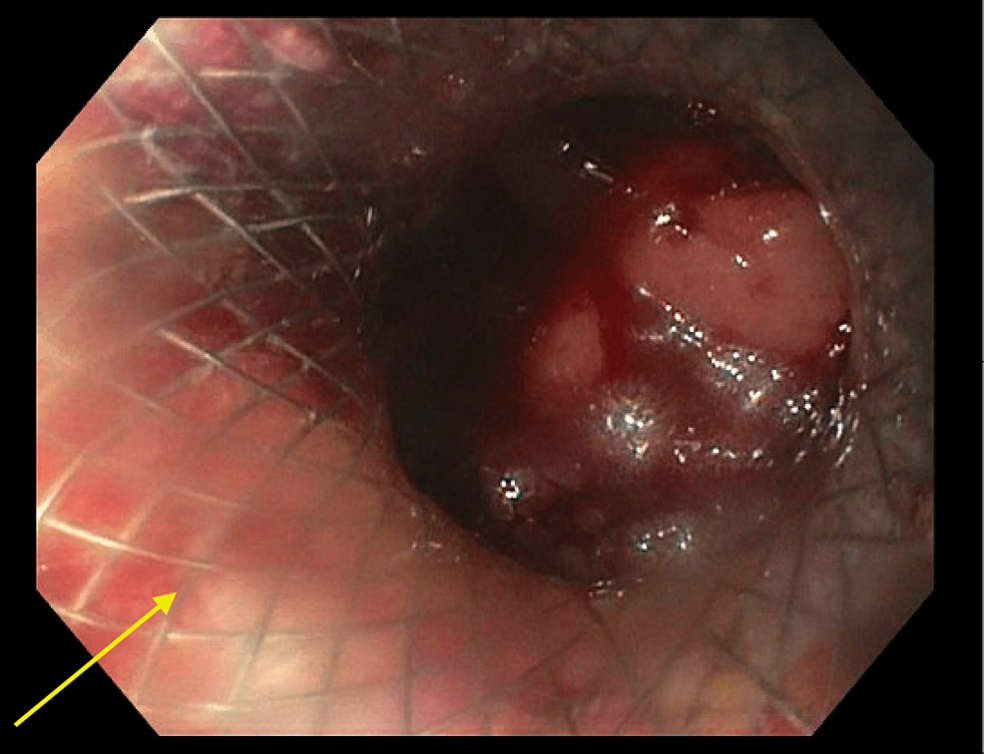
Duodenojejunostomy performed with esophagogastroduodenoscopy (EGD) and endoscopic ultrasound (EUS) with AXIOS stent placement (yellow arrow)

**Figure 3:**
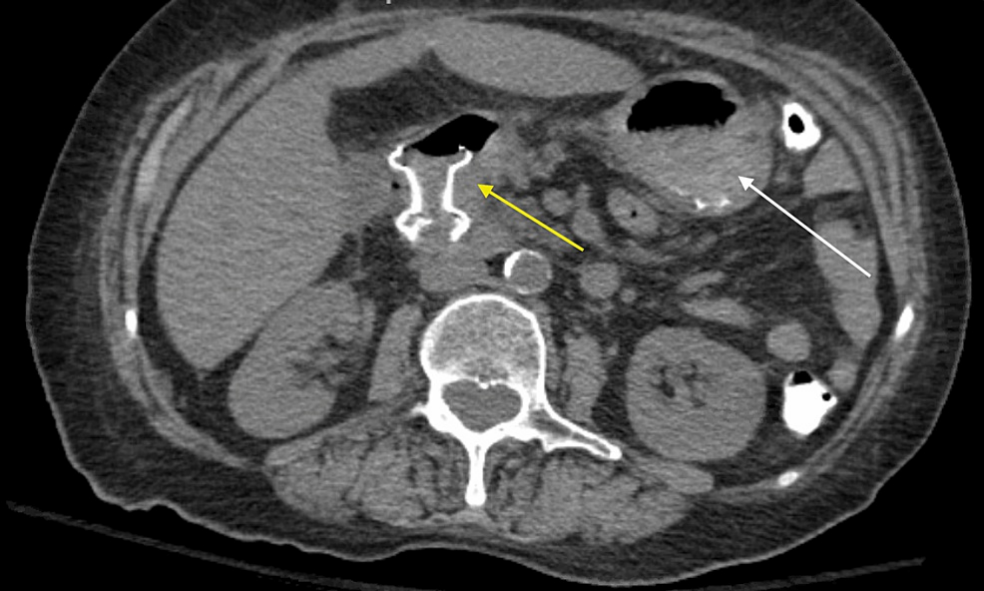
Computerized tomography (CT) of the abdomen and pelvis without contrast demonstrating interval placement of a stent (yellow arrow) with decreasing gastric distension (white arrow)

An esophagogastroduodenoscopy (EGD) was performed; however, there was difficulty passing the obstruction with both adult and pediatric scopes. Ultimately, a decision was made to create a duodenojejunostomy to relieve the duodenal obstruction using the AXIOS stent (Boston Scientific Corporation, MA, USA) system. A 0.035-inch x 450-centimeter straight Hydra Jagwire (Boston Scientific Corporation, MA, USA) was inserted through the needle under fluoroscopic guidance. The AXIOS stent and electrocautery device were introduced through the working channel and advanced over the guidewire. The AXIOS device was advanced into the cyst, and a 20 x 10-millimeter AXIOS stent was placed in close approximation to the walls of the duodenum and the jejunum (Figure 2). A subsequent CT scan of the abdomen and pelvis without contrast illustrated the patency of the stent and appropriately decreased the gastric distention (Figure 3).

Given the advanced stage of her malignancy and other comorbidities, a decision was made to focus on palliative measures rather than continue with chemotherapy. By hospital day 4, she was tolerating a regular oral diet and was discharged to hospice care.

## Discussion

Pancreatic cancer remains one of the most fatal malignancies, with a five-year survival rate of 8.5% in the United States [9]. At the time of diagnosis, 29% of patients will have local invasion and 52% will have metastatic disease [9]. Patients are often asymptomatic for a 10-year latency period prior to their diagnosis [11]. Due to this, they present at an advanced stage with complications such as GOO [2]. Although GOO may be treated with specific chemotherapy regimens, surgical resection with gastrojejunostomy is the gold standard of treatment, especially in those with a life expectancy greater than two months [12]. However, there are newer and less invasive approaches using endoluminal stenting [13]. Endoluminal stenting has been traditionally used to relieve biliary obstruction since the 1980s; however, since the early 2000s, metal endoluminal stents have been used for the palliative relief of GOO caused by both pancreatic and inoperable gastric carcinoma, too [3,14-15]. Prior studies have compared endoluminal stenting to gastrojejunostomy and have demonstrated not only efficacy in reducing gastric distention and improving the GOO score but also illustrated decreased morbidity from fewer adverse post-operative complications, shorter hospitalizations, and smaller hospital costs [1-2,14,16]. Other studies evaluated the impact endoluminal stenting had on patients’ QoL compared to traditional gastrojejunostomy, and there were no major differences between the two approaches; however, the time to resolution of oral intake improved significantly with endoluminal stenting [14-15]. In a multicenter retrospective study that included 74 patients with malignant GOO, they found that 96% of the patients tolerated oral intake successfully for the remainder of their lives, and 78.4% did not require further intervention [15]. Compared to gastrojejunostomy, endoluminal stenting increased the rate of survival following relief of the GOO, with approximately 75% of patients alive at 35 days following stenting and 25% alive at 156 days [14]. Tumor overgrowth, stent migration, and/or dysfunction were rare complications observed across many studies evaluating the efficacy of stenting versus gastrojejunostomy [1,3,14-15].

A prospective analysis compared the efficacy of covered and metal endoluminal stents in gastric versus pancreatic carcinoma, with the hypothesis that different tumor locations may behave differently with endoluminal stenting [3]. Although stenting was clinically successful and the overall complication rate did not differ between both groups, the incidence of stent collapse was higher in the gastric carcinoma group [3]. Additionally, the risk of serious adverse outcomes, such as gastrointestinal bleeding or perforation, was significantly higher in patients with pancreatic cancer [3]. Patients with GOO secondary to gastric cancer had a longer duration of survival following palliative stenting, with a median survival of 153 days compared to 90 days in the pancreatic cancer group [14].

Although numerous studies report the clinical superiority of endoluminal stenting to gastrojejunostomy, this approach is still not routinely practiced. With endoluminal stenting, palliative measures can be achieved with minimal risk in patients with short life expectancies and who are poor surgical candidates.

## Conclusions

This case raises awareness of the potential of utilizing stent placement for relief of malignant GOO secondary to advanced pancreatic adenocarcinoma. Numerous studies have compared the safety and efficacy of metal endoluminal stents to traditional gastrojejunostomy, and have found superior and/or non-inferior results; however, there are still no clear guidelines or optimal patient demographics to determine the patients who would most benefit from this approach. Studies have shown that patients who are poor surgical candidates and have an Eastern Cooperative Oncology Group performance status of less than two have a longer patient survival rate. More research should be done on this topic, as endoluminal stenting is becoming the preferred approach worldwide.
